# 847. Cryptococcal disease in HIV and non-HIV-infected patients: clinical features and outcome

**DOI:** 10.1093/ofid/ofad500.892

**Published:** 2023-11-27

**Authors:** Carla M Roman-Montes, Mariana Velez-Pintado, Lisset Seoane-Hernandez, Andrea Rangel-Cordero, Rosa Areli Martínez-Gamboa, Brenda Crabtree-Ramírez, Vida V Ruiz-Herrera, Jose Sifuentes-Osornio, Alfredo Ponce-de-Leon, Fernanda González-Lara

**Affiliations:** INCMNSZ, Mexico, Distrito Federal, Mexico; Instituto Nacional de Ciencias Médicas y Nutrición Salvador Zubirán, Mexico City, Distrito Federal, Mexico; INCMNSZ, Mexico, Distrito Federal, Mexico; INCMNSZ, Mexico, Distrito Federal, Mexico; INCMNSZ, Mexico, Distrito Federal, Mexico; INCMNSZ, Mexico, Distrito Federal, Mexico; University of Guadalajara, Guadalajara, Jalisco, Mexico; INCMNSZ, Mexico, Distrito Federal, Mexico; INCMNSZ, Mexico, Distrito Federal, Mexico; INCMNSZ, Mexico, Distrito Federal, Mexico

## Abstract

**Background:**

Cryptococcosis is an emerging fungal infection, particularly in patients without HIV. Yet, late diagnoses and worse prognoses are seen. We aimed to compare clinical characteristics and outcomes in patients with cryptococcosis.

**Methods:**

We conducted a retrospective cohort (2001-2021) of patients with cryptococcosis, comparing HIV+ and non-HIV groups in a tertiary center in Mexico. The primary outcome was 20-week mortality. We used descriptive statistics and logistic regression for mortality.

**Results:**

We found 107 patients, 51.5% (55) in HIV+ and 48.5% (52) non-HIV. HIV+'s median CD4+ count was 25 (IQR 12-47). In the non-HIV group, patients were older (median 53.5 vs. 40 years, p=< 0.02), less frequently male (40% vs. 89% p=0.01), and more with autoimmune diseases (41% vs. 0%, p= < 0.01) were found; only 6% were immunocompetent.

The most frequent presentation was disseminated (51% vs. 35%, p=0.08), followed by meningeal (38% vs. 33%, p=0.5). The pulmonary disease was frequent in non-HIV group (27% vs. 4% p=0.001). The most frequent isolated species was *Cryptococcus neoformans* (89% HIV+ vs. 98% non-HIV group, *p*=0.06), followed by *C. neoformans var grubii* (9% HIV+ vs. 2% non-HIV *p*=0.10) and *C. gattii* (2% HIV+ *p*=0.32) and was most frequently isolated from cerebrospinal fluid (CSF) in the HIV+ (64% vs. 38% p=0.009). Serum cryptococcal antigen was less frequently positive in the non-HIV group (44% vs. 88% HIV, p=0.007). The median days from symptom onset to diagnosis were longer in HIV+ (25 vs. 7, p< 0.01).

Both groups received similar antifungal treatment (87% vs. 94% HIV, p=0.16). No difference in mortality in both groups (31% vs. 31% p=0.9). In multivariate analysis, diabetes mellitus (DM) was associated with increased mortality (OR 6.9 (95%CI 2.1-22.7), while antifungal treatment was associated with reduced mortality (OR 0.11 95%CI 0.02-0.59).

**Table 1.**
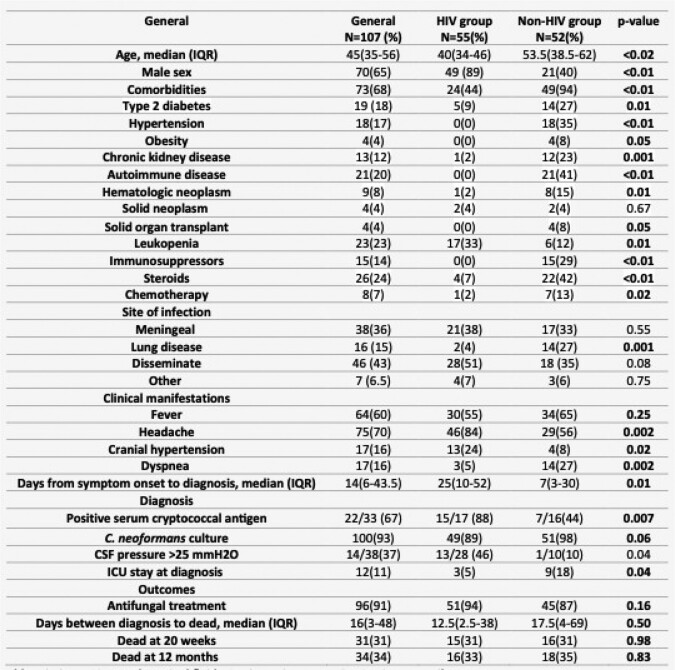
General characteristics in Cryptococcal disease in HIV and non-HIV infected patients. Abbreviations. CSF: cerebrospinal fluid, ICU: intensive care unit, IQR: interquartile range.

**Conclusion:**

In this retrospective cohort, non-HIV with Cryptococcosis were mostly females with autoimmune diseases and pulmonary involvement, compared with younger males with cryptococcal meningitis among HIV+ patients. A late presentation was frequent in HIV+. We found no difference in mortality at 20 weeks between both groups. DM was associated with increased mortality, and the proportion of patients with DM and a worse outcome is a relevant finding.

**Disclosures:**

**All Authors**: No reported disclosures

